# Total Delay in Treatment of Tuberculosis and Associated Factors among New Pulmonary TB Patients in Selected Health Facilities of Gedeo Zone, Southern Ethiopia, 2017/18

**DOI:** 10.1155/2019/2154240

**Published:** 2019-06-02

**Authors:** Netsanet Awoke, Bedado Dulo, Feven Wudneh

**Affiliations:** Department of Medical Laboratory Science, Dilla University, P.O. Box 419/13, Ethiopia

## Abstract

**Background:**

TB is an infectious disease caused by the bacillus* Mycobacterium TB *complex. It is a major public health concern causing devastating illness in millions of people each year and one of the top 10 causes of death worldwide following HIV pandemic. It demands huge costs each year for prevention, diagnosis, and treatment of TB. Global TB control progress depends on major advances in early diagnosis and treatment. Despite progress in providing diagnosis and preventive treatment of TB, big detection and treatment gaps remained with delayed diagnosis and treatment of TB especially in resource-limited countries. This is mainly because of factors related to the patient and health care system including sociodemographic, economic, and cultural barriers to accessing TB care.

**Objective:**

The study conducted in Gedeo Zone, Southern Ethiopia, had the primary purpose of identifying the median delay in starting a correct TB treatment and the associated factors for such a delay in patients newly diagnosed with PTB in selected health facilities of Gedeo Zone, Southern Ethiopia, 2017/18.

**Methods:**

Institutional based cross-sectional study was conducted among new pulmonary TB patients in selected health institution of Gedeo Zone, Southern Ethiopia, 2017, from October, 2017, to May, 2018. All new pulmonary TB patients who fulfill the inclusion criteria during the study period were included in the study after informed consent was obtained from the participants. Data was cleaned, coded, and entered into SPSS version 20 for analysis. A frequency for variables was calculated. Chi-square was used to screen the possible potential associated factors and multivariate analysis was used to ascertain the association between variables. All statistical tests values of p<0.05 were considered as statistically significant.

**Result:**

The median total in treatment of TB was 60 days. Among the total study participants, 50.9% of the participants have unacceptable/longer total delay in TB treatment. Being of female gender, not attending formal education, having rural residency, having poor knowledge of TB, having home distance >10Km from the nearest health facility, visiting nonformal health care provider, and taking antibiotic treatment before TB diagnosis empirically were identified as significant independently associated factors for unacceptable total treatment in TB.

**Conclusions:**

There was higher median total delay in treatment of TB (60 days) and an overall prevalence of 50.9% unacceptable/longer total delay in treatment of TB. Female gender, rural residence, not attending formal education, visiting nonformal health facility as first health care seeking, having poor knowledge of TB, and having antibiotic treatment before TB diagnosis were identified as independent significant associated factors.

## 1. Introduction

Tuberculosis is an infectious disease caused by the bacillus* Mycobacterium TB *complex [1]. TB typically affects the lungs (pulmonary TB) but can also affect other sites (extra-pulmonary TB) including the meninges, kidneys, bones, and lymph nodes. It is most commonly transmitted via minute aerosol droplets of the bacilli that remain suspended in the air for prolonged periods of time, posing a particular infection-control challenge [1, 2].

The most commonly reported symptom of pulmonary TB is persistent (2 weeks or more) cough generally, but not always, with or without sputum production, chest pain, and sometimes blood (hemoptysis) [3]. In persons with TB, the cough is often accompanied by systemic symptoms such as fever, blood tagged sputum, night sweats, and weight loss [4]. Clinical manifestations of TB are dependent on a number of factors: age, immune status, coexisting diseases, immunization status to the bacillus, virulence of the infecting organism, and host-microbe interaction [1, 5].

Tuberculosis is a major public health concern worldwide, despite having a slow decline in incidence over the last decade [1]. Control of TB in the community depends on early diagnosis and treatment which is the key elements of the TB control program. Patients suspected with TB should be diagnosed and initiate treatment as early as possible. This is because of its importance for containing the spread of the disease within the community and for effective TB management [3].

The identification of TB cases depends on patients recognizing TB symptoms and seeking appropriate health care as well as the capacity of the health care system to early diagnose the disease. Missed opportunities for earlier detection and treatment of TB lead to increased disease severity for the patients [6] and a greater likelihood of transmission of M. tuberculosis plus multidrug resistance tuberculosis (MDR-TB) to family members and others in the community [4]. In addition, it may avert early mortality associated with more serious complications and severe disease due to delayed presentation [7]. This is because the majority of TB transmission occurs between the onset of cough and initiation of treatment, and patients become more contagious and result in ongoing transmission as the delay increases [2, 7].

Prevention of new infections of* Mycobacterium tuberculosis *and their progression to tuberculosis (TB) disease is critical to reduce the burden of disease and death caused by TB. Ensuring widespread patient access with fast turnaround time and high-quality diagnosis and early treatment is required for effective TB control [2]. In regions where high prevalence of TB, an early diagnosis is considered as one that is performed between 2-3 weeks after the onset of TB clinical symptoms and a late diagnosis is that performed 4 weeks after the onset of symptoms which is unacceptable diagnosis delay and is not recommended for effective TB control [8].

Currently, although sputum smear microscopy remains the most widely available test to establish a microbiological diagnosis, other more sensitive means of identifying* M. tuberculosis*, particularly culture and rapid molecular tests, are rapidly gaining acceptance as their performance and applicability are increasingly understood [1, 4]. To create access to the diagnostic and treatment services for MDR-TB, TB culture and drug-susceptibility test laboratories as well as MDR-TB treatment centers have expanded globally, including Ethiopia [2, 9].

In 2016, despite the increases in notifications of TB globally, big detection and treatment gaps remain. This may be due to underdiagnosis or delayed diagnosis and initiation of treatment because of barrier to accessing TB diagnostic and treatment service [6]. Delay in diagnosis and treatment of TB/total treatment delay is mediated by factors related to the patient and health care system [10]. Sociodemographic, economic, and cultural barriers to accessing TB care such as limited knowledge about signs and symptoms of TB, low health seeking behavior of the patients, complex diagnostic procedures, poor diagnosis with long patient waiting time, and difficulty of disease management in health facilities were reported as delay in treatment [2, 7, 10]. So, to fill this TB control gap, in 2015 WHO launched a new global TB strategy “End TB Strategy by 2035”. To achieve the milestones of End TB Strategy by 2035, one of the pillars is integrated early diagnosis and treatment of TB including systematic screening of contacts and high-risk groups and universal drug-susceptibility testing plus early initiation of treatment for all people with TB supported with intensified research [2].

Despite the findings of previous studies investigating the same topic, the study describes the situation of a specific setting, thus strongly related to its particular situation. The importance of the findings is mainly in the possibility of describing the situation of an area where TB is still a high prevalence disease, with a significant risk of underestimation or incorrect treatment. So, the study had primary purpose of identifying the median delay in starting a correct TB treatment and the associated factors for such a delay in patients newly diagnosed with PTB patients in selected health facilities of Gedeo Zone, Southern Ethiopia.

## 2. Materials and Methods

The study was conducted among new pulmonary TB patients in 8 selected health facilities (DURH, Yirgacheffe, Wonago, Gedeb, Bulie, Chelelektu, Chichu, and Harrorissa) of Gedeo Zone, Southern Ethiopia, from October, 2017, to May, 2018.

Institutional based cross-sectional study was conducted among* 422 *patients confirmed with tuberculosis infection in selected health facilities of Gedeo Zone who fulfill the inclusion criteria during the study period. Study participants of ≥15 years old & those new smear positive pulmonary TB patients who transferred into DOTS service of the selected health facilities were included. Study participants who were extra-pulmonary TB patients, ≤15 years old, who was critically ill and could not respond to the interview & who was transferred out to other health facilities for DOTS were excluded.

The health facility was selected based on the TB case report of the zone to fulfill the sample size with the specified data collection period. The total sample size was proportionally allocated based on its case report. The study participants, newly diagnosed as PTB patients, were consecutively recruited in each health center of TB clinic within the specified time period until the intended sample size was met. Number of patients to be interviewed from each health facility was based on the case load report or proportionately allocated to the selected health facilities based on their estimated annual flow of smear positive PTB patients.

## 3. Operational Definitions

*Total delay in treatment*: the time interval between the date onsets of major TB symptoms to first start of anti-TB treatment; in this case if the total delay in treatment is ≥ median total treatment, delay is considered longer/unacceptable delay.

*New PTB*: patients with two or more sputum smears/gene x-pert positive for acid fast bacilli and who had never received treatment for TB for as long as one month.

*Nonformal health providers*: these include traditional health providers (such as traditional injectors/healers, herbalists, spiritual/holy water).

*Current smoker*: an adult who has smoked 100 cigarettes in his or her lifetime and who currently smokes cigarettes. Beginning in 1991 this group was divided into “everyday” smokers or “somedays” smokers.

*Former smoker*: an adult who has smoked at least 100 cigarettes in his or her lifetime but who had quit smoking at the time of interview for the last 2 months.

*Never smoker*: an adult who has never smoked, or who has smoked less than 100 cigarettes in his or her lifetime.

*Stigma*: for each stigma question a score of 1= strongly disagree, 2= disagree, 3= agree, 4= strongly agree response was given. Those with a total score below the ≤25th percentile value of the total question's were classified as having no/low stigma, those with score between 25th and 75th percentile value of the total question's were considered as having moderate stigma, and those with ≥75th percentile value of the total question's were considered as having sever stigma.

*Alcoholic drinker*: patients who drink presently or previously 5% alcohol with more than 500ml per days regularly.

*Knowledge status:* it is the knowledge of the patient regarding the means of transmission, symptoms, and prevention of TB. For each TB knowledge questions a score of one was given for yes response/for the correct answer and a score of zero was given for no response/for the incorrect answer. Then, total knowledge score and median were calculated. The scale was then dichotomized into two categories (good/below the median or poor knowledge equal or above the median) using the median as the cut-off.

*Patient satisfaction*: it is the previous satisfaction of study participants on health care provider and health care system. For each satisfaction question a score of 1= very low, 2= low, 3= medium, 4= good, or 5= very good response was given. Then a separate summary indicator was developed for analyses by dividing the total score into three strata, ≤25th percentile, between 25th and 75th percentile, and ≥75th percentile to label it as good, medium, and low, respectively.

*Formal private health provider*: private drug store, pharmacy, and private clinic opened with official licensed individuals.

*Unacceptable/longer total treatment in TB*: those who has total treatment delay greater than or equal to the median total treatment in TB (*Asefa and Teshome, 2014; Yimer, Bjune and Holm-hansen, 2014; Mandi and Pradesh, 2012; Zeleke and Trifa, 2014*).

## 4. Data Collection and Instruments

Study participants were informed clearly about the purpose of the study. A semistructured questionnaire (translated in English and to the local language) was prepared to interview patients; with this method, the investigators easily extracted data needed for the purpose of the study. Pretest was done on 5% of the sample size in none selected health facilities. The pretested questionnaire was used to collect information from study participants. Before the actual data collection process started, all data collectors (TB clinic nurse or health officer of respective health facilities and supervisors) was given two-day onsite training by the principal investigator on the purpose of the study and how to select study participants and fill the data collection tool. All eligible study participants were interviewed through face-to-face by trained data collector of the TB clinic in the selected health facilities just right after commencing treatment.

The questionnaire and the interviewers were supervised regularly throughout the duration of the study. The questions were open or close ended. For each patient, the following information was collected: sociodemographic characteristics, knowledge, and experience of stigma to TB.

Study participants also were asked to estimate the duration of experiencing pulmonary symptoms in days before first visit to the health facility (patient delay). In addition, they were asked the duration in days from first contact to healthcare providers until TB treatment initiation (health system delay). Finally, they were also asked to estimate the time in days they had been experiencing major pulmonary symptoms before presenting to health facilities with the current diagnosis of TB (total treatment delay). Moreover, they were asked about places they sought for treatment before first visit to the health facility, reasons for delay before visit to the health facility, and distance and time taken from their residence to the health facility.

Local religions and political and agricultural events were used as a calendar to collect information for those patients who were unable to estimate the date of onset of first presenting symptoms for the current diagnosis (TB).

## 5. Data Analysis and Interpretation

Data was checked for completeness and consistency in semistructured questionnaires. Then it was cleaned, coded, and entered using SPSS 20 for analysis. Frequency and cross-tabulation were checked to monitor data quality. Descriptive statistics was used to generate frequencies of categorical variables and summarize data. Tables were used for data presentation. Odds ratio with 95% CI was used to screen the possible associated factors.

Variation in categorizing of total treatment delay of TB has been documented. For the outcome variable, we used the median delay as a cut-off to dichotomize the TB patients into two groups (≥ median versus < median). The median of total delay was categorized as acceptable/shorter or unacceptable/longer total treatment delay. A patient for whom anti-TB treatment was not initiated within the median total treatment delay was considered to experience unacceptable total treatment delay [3, 11–16]. Identified variables were associated with experience of total treatment delay; P value ≤ 0.25 in bivariate analysis was candidate for final logistic analysis model. Multivariate analysis was used to ascertain the association factors. All statistical tests values of p<0.05 were considered as statistically significant.

## 6. Ethical Consideration

The study was approved by Dilla University, College of Health Sciences and Medicine, Research, Dissemination and Community Services Director Office (RDCDO). Ethical clearance was obtained from ethical review committee of Dilla University, College of Health Sciences and Medicine. Letter of support from RDCDO was delivered to selected health facilities administrators. Permission to undertake the research was obtained from the selected health facilities administrators. Moreover, the purpose of the study and benefits of the survey were explained for each study subject before data collection and written consent were obtained from each study participant. Confidentiality of information and privacy of the study participants were also maintained with interviewing each study participants in a separate place and coding to label individual respondents.

## 7. Quality Assurance

To ensure data quality, two-day training for data collectors was given before the start of data collection. The questionnaire was pretested in 5% of the sample size in none of selected health facilities in the zone. The investigators and supervisors followed data collection process regularly during the data collection to monitor overall activities of the data collection, to obtain the filled questionnaire and check for its completeness.

## 8. Results

### 8.1. Sociodemographic Characteristics of the Study Participants

Of the 422 eligible study participants, 235(55.7%) were males with a male to female ratio of 1.3 to 1. The mean age of study participants was 33 years, ranging from 15 to 70 years. Study participants with age group [14–24] years were 31.3%. Of the total study participants, 41.7%, 58.5%, 46.2%, and 40.8% of them were not attending formal education, having rural residency, being farmer, and having ≤500 mean monthly family income, respectively (Table 1).

### 8.2. Clinical Profiles of the Study Participants

In this study, 8.5% of the study participants had previous history of TB and 26.5% of them were house/bed bound because of the current illness (Table 2).

### 8.3. Behavior & Knowledge Related Profiles of the Study Participants

In this study, 14.9% of the study participants were current cigarette smoker and 44.5% of the study participants had poor knowledge of tuberculosis (Table 3).

### 8.4. Health Accessibility & Health Seeking Behaviors of the Study Participants

Out of total study participants, 45% travel >10 Km to the nearest health facility from their home. 26.3% of the study participants visited nonformal health provider for consultation initially because of the illness. Similarly, 26.3% of the study participants visited nonformal health care provider (Table 4).

### 8.5. Total Treatment Delay in TB

**Figure 1:**
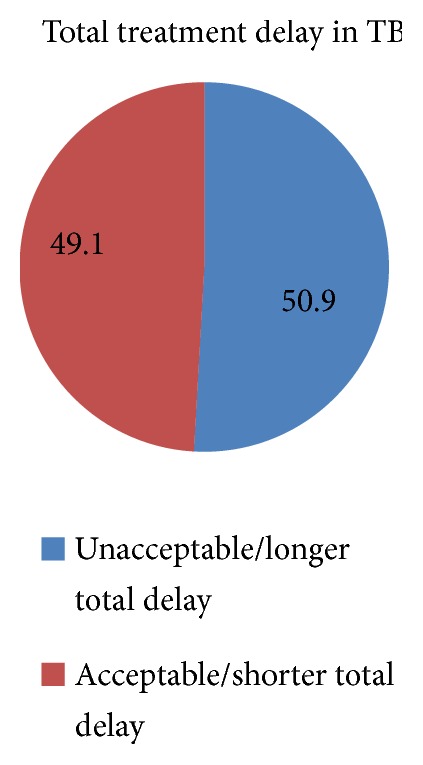
Total treatment delay in TB.

In this study, the median total delay in treatment of TB was 60 days (IQR: 33-81), [95% CI, 42-63]. Of the total study participants, 215 (50.9%) [95% CI, 45.4-55.7] of the participants have unacceptable/longer total delay in TB treatment (delay > 60 days) (Figure 1).

### 8.6. Distribution of Total Delay in TB Treatment among Sociodemographic Characteristics

Being female gender (67.9%) among sex, age of > 45 years old (55.8%) among age group, not attending formal education (78.4%) among educational status, having rural residence (66.0%) among place of residence, and having mean family size of >7 individuals (62.2%) among mean family size of the study participants had higher unacceptable total delay in treatment of TB (Table 5).

### 8.7. Distribution of Total Delay in Treatment among Clinical Profiles

In this study, not taking TB treatment previously (51.8%), being house/bed bound (71.4%) had higher unacceptable total delay in treatment of TB among presence of previous history of TB & level of severity of the illness before first visit to health facility respectively (Table 6).

### 8.8. Distribution of Total Delay in Treatment of TB among Behavior & Knowledge Related Profiles of the Study Participants

In this study, current smoker (57.1%) among smoking status, poor knowledge of TB (64.4%), and low patient satisfaction (58.3%) among patient satisfaction to healthcare system had higher unacceptable total delay in treatment of TB (Table 7).

### 8.9. Distribution of Total Treatment Delay among Health Accessibility & Health Seeking Behaviors of the Study Participants

Having home distance >10km (69.5%) among home distance from current health facility, visiting nonformal health provider (82.0%) among first health care seeking behavior, and visiting ≥3 healthcare providers (72.9%) among number of healthcare providers visiting had higher unacceptable total delay in treatment of TB (Table 8).

### 8.10. Bivariate and Multivariate Analysis of Associated Factors for Longer Total Treatment Delay

In univariate analysis, sex, education status, residency, family size, marital status of study participants, monthly family income, level of severity before first visit to health facility, presence of previous history of TB, stigma, knowledge of TB, home distance from the nearest health facility, presence of other health facilities close to home, first health care seeking behavior, number of healthcare providers visiting and presence of antibiotic treatment because of current illness had p<0.25 which was eligible for multivariate analysis.

In multivariate analysis, after adjusting the eligible variable, only being of female gender, not attending formal education, having rural residency, having poor knowledge of TB, having home distance >10km from the nearest health facility, visiting nonformal health care provider, and taking antibiotic treatment because of current illness was identified as independently associated risk factors for unacceptable total delay in treatment of TB as presented in Table 9.

## 9. Discussion

Early diagnosis and initiation of treatment is a determining factor for spread and infectivity of TB. This may worsen the disease and cause higher mortality which is still prevailing problem around the globe especially in resource-limited countries [2, 3, 6, 17]. The present study focuses on assessing the total delay in TB treatment & associated factors among patients with pulmonary tuberculosis. Variations of total delay in treatment of TB were reported from setting to setting across countries because of geographical, economic, and cultural variations to access TB diagnosis and treatment service [3, 18]. This study identified that median total delay in treatment of TB was 60 days (95% CI, 42-63). This is consistent with a study done in Uzbekistan [11]; Southern Ethiopia [25]; Northwest Ethiopia [16]; and West Gojjam, Ethiopia [17], with a median total treatment delay of 50 days, 45 days, 60 days, and 60 days, respectively.

This finding is higher than that of study done in India [15]; Uganda [12]; Nepal [19]; Arsi Zone, Ethiopia [20]; and Arbaminch, Ethiopia [21], with a median total treatment delay of 36 days, 28 days, 39.5 days, 40 days, and 35 days, respectively. This difference may be due to socioeconomical difference of access to TB care (available diagnostic facilities), inclusion/exclusion criteria, and sample size. Another reason might be difference in study area/population difference (health seeking behavior, knowledge and attitude of the participants).

This finding is lower than that of study done in Ghana [22]; Guinea-Bissau [6]; Italy [23]; Ahvaz [24], Bale Zone Ethiopia [14]; East Wollega, Ethiopia [26]; Amhara region, Ethiopia [27]; Afar region, Ethiopia [28], with a median total treatment delay of 104 days, 84.7 days, 77.5 days, 73 day, 97 days, 90 days, 80 days, and 70.5 days, respectively. This difference may be due to difference in access to TB care (available diagnostic facilities), inclusion/exclusion criteria, sample size, study area/population difference (health seeking behavior, knowledge, and attitude of the participants), and study period (improvement in diagnosis, treatment, and monitoring services considerably in the last five years may improve the delay in treatment. This might be due to expansions of DOTS services, which made better accessibility in seeking health care for TB treatments compared to the previous study). Another possible reason might be that this study includes some urban and most rural health facilities compared to a solely rural setting in Guinea-Bissau [6].

In the present study, it was found that there was an overall 50.9% (95% CI, 45.4-55.7) prevalence of unacceptable total delay in treatment of TB. This observed result is in line with a previous study in Uzbekistan [11]; Arsi Zone, Ethiopia [20]; Southern Ethiopia [25]; Afar region [28]; Arbaminch, Ethiopia [21]; and West Gojjam, Ethiopia [17], with 50%, 48.9%, 49%, 50%, 46%, and 50% of patients with observed unacceptable delay, respectively.

This finding is higher than that of a study done in India [15] with 29% of patients with observed unacceptable delay. However, it is lower than study done in Ghana [22], Uganda [12], and Nepal [19], with 82.2 %, 91%, and 62.8 % of patients with observed unacceptable delay, respectively. This discrepancy might be due to differences in sample size, study setting, and dichotomization of cut-off points of total treatment delay as cut-off points to determine delays.

Understanding the factors associated with patient delays is vital for the achievement of reduction in TB morbidity and mortality. Studies conducted in different countries have shown that many factors are involved in delayed treatment of tuberculosis patients [3, 10, 29]. In the study, being of female gender was identified as independent associated factor for development of unacceptable total delay in treatment of TB than their counterparts. This is similar with study in Ahvaz [24], Nigeria [13], Uganda [12], and Southern Ethiopia [25]. This may be due to the fact that socioeconomic and cultural position of women may influence their opportunities and add constraints to healthcare access and needs [30]. This may be explained by limited decision making power, engagement in domestic work, unemployment, and facility too far in study area [30–32].

Regarding educational status, not attending formal education was reported as associated factor for development of unacceptable total delay in treatment of TB in this study. This is in line with study done in Guinea-Bissau [6], Mediterranean region, Egypt [3], and Ethiopia [33]. This could be due to the fact that those attending tertiary education might have better information about TB, increased awareness of the TB disease, and being reinforced by good treatment seeking behavior and may likely seek care early [34]

Having rural residency among the study participants was also reported as associated factor for unacceptable total delay in treatment of TB development in this study. This is similar with study done in Mediterranean region, Pakistan and Somalia [3], Nigeria [13], and three studies from Ethiopia (10 DOTS districts of Ethiopia [33], North Wollo zone [35], and Arbaminch [21]). This may be explained such that rural residency may limit the study participants to have appropriate access to health information and health facilities than the urban residency. In rural residency they may have lack of supervision of health staff to provide health information at peripheral level resulting in low education level of TB than urban areas [34]. This may predispose the individual to have poorer access to health care in rural areas. This is because of the fact that rural residency may make it difficult for the participants to travel to health facility in terms of travel time from patient's areas of residences to public facilities which is closely related to income level [34, 36, 37].

Moreover, home distance to travel > 10 km to reach the nearest public health facility was also reported as causing unacceptable delay in TB treatment development. This finding is consistent with study in Mediterranean region, Egypt and Yemen [3], Nigeria [38], Bale Zone, and Ethiopia [14]. This may be because the facility is too far from the study participant's home which may limit access to health care in terms of travel time from patients' areas of residences to public health facilities [37, 39]. This may result in low accessibility in health service & delay in seeking health care of TB treatments.

Furthermore, health care seeking behaviors of the respondents were documented for unacceptable treatment delay in TB in this study. Visiting nonformal health provider was identified as associated factor for development of unacceptable total delay in treatment of TB. This is in line with study in Uzbekistan [11], India [15], Mozambque [38], Afar, Ethiopia [28], Mediterranean region, Somalia [3], three studies from Ethiopia (Amhara region [27], West Gojjam [17], North and Wollo zone [35], Bale Zone, Ethiopia [14]). This might cause low reliance of treatments in health facility and strong cultural bond in holy water and traditional healer treatments leading the respondents to seek care for TB treatments after failure of treatments in informal sources [40].

With respect to knowledge of the study participants, this study revealed that having poor knowledge of TB is an associated factor in development of unacceptable total delay in treatment of TB. This finding is consistent with study documented in Mediterranean region, Pakistan [3], India [15], Mozambque [38], North Wollo, Ethiopia [35], and West Gojjam, Ethiopia [17]. This might be due to study participants with good knowledge about TB might have better inclination for early seeking of medical care. This means having good knowledge of tuberculosis means of transmission, prevention diagnosis & treatment option are vital for early seeking of TB medical care.

Prior unspecific treatment before diagnosis of the current illness was identified as associated factor for delay in TB treatment in this study. Similar studies were reported in Italy [23]. Patients receiving antibiotics prior to TB confirmation experience a process-related delay in starting treatment. This empirical antibiotic treatment may lower some sign/symptoms of TB which will make the patient delay to request diagnosis & treatment [41]. For example, fluoroquinolones treatment was found to be effective in lower respiratory symptoms of TB and initial results usually improvement. This may suggest the delay was due to the time inherent in taking a course of antibiotics and waiting to see if there is a clinical response [42, 43].

## 10. Limitations of the Study

- The design of our study unfortunately did not outline to identify factors such as antibiotic treatment (the prescriber/source of the drug, type drug & duration).
- This study lacks standard cut-off point for unacceptable total treatment delay and we used the cut of point as the median of the total treatment delay.
- Furthermore, information for this study relied on the participants self-reported duration of total delay and therefore it may be subject to recall bias.

## 11. Conclusions

It revealed that there is still a high median total delay (60 days) with an overall high prevalence of unacceptable/longer total delay of 50.9%. Female gender, rural residence, not attending formal education, visiting nonformal health facility as first health care seeking, having poor knowledge of TB & having antibiotic treatment before the current TB diagnosis were identified as independent associated factors for unacceptable total delay in treatment of TB.

## 12. Recommendations

Woreda, Zonal & other health stakeholders office collaborative work should be developed to further decentralization of TB diagnostic and treatment facilities to the periphery to increase & expand health care accessibility (TB diagnosis & treatment service) among people living in rural areas, especially among females in all areas.

1. Tuberculosis control programmes in similar settings should consider innovative strategies for TB education, advocacy, communication, and social mobilization to the rural area & female gender.
2. Due attention should be given to increase public awareness & health education about the symptoms of tuberculosis, the availability and location of free diagnostic services & seeking early care and treatment of TB with designing health education directed towards how to suspect and recognize TB symptoms and the importance of early health seeking.
3. Woreda, Zonal & other health stakeholders offices should give emphasis to collaborate with informal health care provider (traditional healers, local drug sellers, traditional healers, and religious leaders) to design training & discussion on how to suspect TB symptoms & the way to refer early to government health facilities for diagnosis.
4. Woreda, Zonal & other educational stakeholders should work on to increase & expand accessibility of formal education to the community for early diagnosis & treatment of TB.
5. The duration of total delay and the significant factors of the delay identified in the present study should be incorporated into routine TB surveillance report protocol.
6. Physicians should exert efforts to minimise prior antibiotic treatment of respiratory tract infection by including TB in their differential diagnosis and initiate simple, rapid relevant TB-focused diagnostic investigations early on in the diagnostic process to quickly exclude* M. tuberculosis *in the management of respiratory tract infection.
7. Further nationwide and extensive surveillance on duration of total delay and the significant factors of the delay should be done to design & develop programme alternative diagnosis & prevention, & treatment strategies.

## Figures and Tables

**Table 1:** Sociodemographic characteristics of the study participants among selected health facilities of Gedeo Zone, Southern Ethiopia, 2017/18.

Variables	Frequency (N= 422)	Percentage (%)
*Sex of the participants*		
Male	235	55.7
Female	187	44.3

*Age of the participants/ year*		
15-25	132	31.3
26-35	125	29.6
36-45	104	24.6
>45	61	14.5

*Marital status of patients*		
Married	234	55.5
Single	170	40.3
Others	18	4.3

*Educational status/level*		
Not attended formal education	176	41.7
Primary school	144	34.1
Secondary school	70	16.6
Higher education	32	7.6

*Religion of the study participants*		
Orthodox Christian	94	22.3
Protestant Christian	272	64.5
Muslim	56	13.3

*Study participants place of residence*		
Urban	175	41.5
Rural	247	58.5

*Ethnicity of the study participants*		
Gedeo	247	58.5
Oromo	82	19.4
Amhara	36	8.5
Sedama	39	9.2
Others	18	4.3

*Occupation of the study participants*		
Farmer	195	46.2
Employed	56	13.3
Daily laborer	45	10.7
Student	77	18.2
Merchant	49	11.6

*Mean family size of the study participants*		
1-4 individuals	146	34.6
5-7 individuals	202	47.9
>7 individuals	74	17.5

*Estimated mean monthly family income /ETB*		
≤500	172	40.8
501-1000	122	28.9
>1000	51	12.1
Unknown/no- regular income	77	18.2

**Table 2:** Clinical profile of study participants among selected health facilities of Gedeo Zone, Southern Ethiopia, 2017/18.

Variables	Frequency (N=422)	Percentage (%)
*Presence of previous history of TB*		
Yes	36	8.5
No	386	91.5

*Level of severity before first visit to health facility related to his daily activity*		
Still do a full day work	121	28.7
Some activity outside the house	189	44.8
House/bed bound	112	26.5

**Table 3:** Distribution of behavior related profiles among study participants in selected health facilities of Gedeo Zone, Southern Ethiopia, 2017/18.

Variables	Frequency (N=422)	Percentage (%)
*Smoking status/behaviors of the study participants*		
Never smoke	290	68.7
Former smoker	69	16.4
Current smoker	63	14.9

*Alcoholic intake status/behaviors of the study participants*		
Never drink	209	49.5
Former drinker	130	30.8
Current drinking	83	19.7

*Knowledge score to TB*		
Good	234	55.5
Poor	188	44.5

Stigma score to TB		
No/mild	249	59.0
Moderate	113	26.8
Sever	60	14.2

*Patient satisfaction score to healthcare system*		
Good	175	41.5
Medium	223	52.8
Low	24	5.7

**Table 4:** Health accessibility & health seeking behaviors of the study participants among selected health facilities of Gedeo Zone, Southern Ethiopia, 2017/18.

Variables	Frequency (N=422)	Percentage (%)
*Home distance from the nearest health facility*		
≤10km	232	55.0
>10km	190	45.0
*Presence of other HF close to home*		
Yes	175	41.5
No	247	58.5
*First health care seeking/consultation action because of the illness*		
Gov.t health care provider	159	37.7
Formal private health care provider	152	36.0
Nonformal health care provider	111	26.3
*Number healthcare providers visited because of current illness*		
≤2 healthcare providers	282	66.8
≥3 healthcare providers	140	33.2
*Antibiotic Rx because of current illness before diagnosis made*		
Yes	219	51.9
No	203	48.1

**Table 5:** Distribution of total treatment delay among sociodemographic characteristics of the study participants in selected health facilities of Gedeo Zone, Southern Ethiopia, 2017/18.

Variables	Total treatment delay (≥60 days)		
	No (%)	Yes (%)	Total
Sex of the participants			
Male	147 (62.6)	88( 37.4)	235
Female	60 (32.1)	127 (67.9 )	187

*Age of the participants/year*			
15-25	69 (52.3 )	63 ( 47.7)	132
26-35	125 (52)	60 (48 )	125
36-45	46( 44.2)	58 (55.8 )	104
>45	27 ( 44.3)	34 ( 55.7)	61

*Marital status of study participants*			
Married	106(45.3)	128(54.7)	234
Single	106(53.5)	128(46.5)	234
Others	10(55.6)	8 (44.4)	18

*Educational status/level*			
Not attended formal education	38(21.6)	138(78.4)	176
Primary school	99(68.8)	45(31.2)	144
Secondary school	48 (68.6)	22(31.4)	70
Higher education	22(68.8)	10(31.2)	32

*Religion of the study participants*			
Orthodox Christian	46(48.9)	48(51.1)	94
Protestant Christian	132(48.5)	140(51.5)	272
Muslim	29(51.8)	27(48. 2)	56

*Place of residence*			
Urban	123(70.3)	52(29.7)	175
Rural	84(34.0)	163(66.0)	247

*Ethnicity of the study participants*			
Gedeo	120(48.6)	127(51.4)	247
Oromo	34(41.5)	48(58.5)	82
Amhara	20(55.6)	16(44.4)	36
Sedamo	23(59.0)	16(41.0)	39
Others	10(55.6)	8(44.4)	18

*Occupation of the study participants*			
Farmer	92 (47.2)	103(52.8)	195
Employed	28(50.0 )	28(50.0 )	56
Daily laborer	21(46.7 )	24(53.3 )	45
Student	40(51.9)	37(48.1)	77
Merchant	26(53.1)	23(46.9)	49

*Mean family size of the study participants*			
1-4 individuals	77(52.7)	69(47.3)	146
5-7 individuals	102(50.5)	100(49.5)	202
	28(37.8)	46 (62.2 )	74

*Estimated mean monthly family income /birr*			
≤500	70(40.7)	102(59.3)	172
501-1000	72(59 )	50( 41)	122
	28(54.9)	23(45.1)	51
Unknown/ No-regular income	37( 48.1)	40(51.9)	77
* Total *	*207(49.1)*	*215(50.9)*	*100%*

**Table 6:** Distribution of total treatment delay among clinical profiles of the study participants in selected health facilities of Gedeo Zone, southern Ethiopia, 2017/18.

Variables	Total treatment delay (≥60 days)		
	No (%)	Yes (%)	Total
*Presence of previous history of TB*			
Yes	21(58.3)	15(41.7)	36
No	186(48.2)	200(51.8)	386

*Level of severity of the illness before first visit to health facility*			
Still do a full day work	72(59.5)	49(40.5)	121
Some activity outside the house	103(54.5)	86(45.5)	189
House/bed bound	32(28.6)	80(71.4)	112
*Total *	***207(49.1)***	***215(50.9)***	***100%***

**Table 7:** Distribution of total delay in treatment among behavior & knowledge related profiles of study participants in selected health facilities of Gedeo Zone, Southern Ethiopia, 2017/18.

Variables	Total treatment delay (≥60 days)		
	No (%)	Yes (%)	Total
*Smoking status/behaviors of the study participants*			
Never smoke	146(50.3)	144(49.7)	290
Former smoker	34(49.3)	35(50.7)	69
Current smoker	27(42.9)	36(57.1)	63

*Alcoholic intake status/behaviors of the study participants*			
Never drink	102(48.8)	107 (51.2)	209
Former drinker	67(51.5)	63(48.5)	130
Current drinking	38(45.8)	45(54.2)	83

*Knowledge to TB*			
Good	140(59.8)	94(40.2)	234
Poor	67(35.6)	121(64.4)	188

*Stigma score to TB*			
No/mild	132(53.0)	117(47.0)	249
Moderate	47(41.6)	66(58.4)	113
Sever	28(46.7)	32(53.3)	60

*Patient satisfaction score to healthcare system*			
Good	87(49.7)	88(50.3)	175
Medium	110(49.3)	113(50.7)	223
Low	10(41.7)	14(58.3)	24
*Total *	*207(49.1)*	*215(50.9)*	*100%*

**Table 8:** Distribution of total treatment delay among health accessibility & health seeking behaviors of the study participants in selected health facilities of Gedeo Zone, Southern Ethiopia, 2017/18.

Variables	Total treatment delay (≥60 days)		
	No (%)	Yes (%)	Total
*Home distance from the nearest health facility*			
≤10km	149(64.2)	83(35.8)	232
>10km	58(30.5)	132(69.5)	190

*Presence of other HF close to home*			
Yes	113(64.6)	62(35.4)	175
No	94(38.1)	153(61.9)	247

*First health care seeking/consultation action because of the illness*			
Visited gov.t health facility	107(67.3)	52(32.7)	159
Visited formal private health provider	80(52.6)	72(47.4)	152
Visited nonformal health provider	20(18.0)	91(82.0)	111

Number healthcare providers visited			
≤2 healthcare providers	169(59.9)	113(40.1)	282
≥3 healthcare providers	38(27.1)	102(72.9)	140

Antibiotic Rx because of current illness before diagnosis			
Yes	78(35.6)	141(64.4)	219
No	129(63.5)	74(36.5)	203
*Total *	*207(49.1)*	*215(50.9)*	*100%*

**Table 9:** Bivariate and multivariate analysis of associated factors for total treatment delay among study participants of selected health facilities of Gedeo Zone, southern Ethiopia, 2017/18.

Variables	Total treatment delay (≥ 60 days)			Bivariate		Multivariate	
	No (%)	Yes (%)	Total	*COR (95% CI)*	*P- value*	*AOR (95% CI)*	*P- value*
*Sex of the participants*					*0.001∗*		*0.001∗*
Male	147(62.6)	88( 37.4)	235	1		1	
Female	60 (32.1)	127 (67.9 )	187	3.5(2.3-5.3)	0.001	2.6(1.5-4.5)*∗∗*	0.001**∗∗**

*Age of the participants/ year*					0 .475		
15-25	69 (52.3 )	63 ( 47.7)	132	0.9(0.6-1.6)	0.965		
26-35	125 (52)	60 (48 )	125	1			
36-45	46( 44.2)	58 (55.8 )	104	1.4(0.8-2.3)	0.242		
>45	27 ( 44.3)	34 ( 55.7)	61	1.4(0.7-2.5)	0.322		

*Marital status of study participants*					0.225^**∗**^		
Married	106(45.3)	128(54.7)	234	1			
Single	106(53.5)	128(46.5)	234	0.7(0.5-1.0)	0.103		
Others	10(55.6)	8 (44.4)	18	0.7(0.3-1.7)	0.403		

*Educational status/level*					0.001**∗**		0.001**∗∗**
Not attended formal education	38(21.6)	138(78.4)	176	7.9(3.5-18.3)	0.001	7.0(2.5-19.7)	0.001**∗∗**
Primary school	99(68.8)	45(31.2)	144	1.0(0.4-2.3)	1.000	1.1(0.4-2.9)	0.891
Secondary school	48 (68.6)	22(31.4)	70	1.0(0.4-2.5)	0.986	1.2(0.4 3.5)	0.774
Higher education	22(68.8)	10(31.2)	32	1		1	

*Religion of the study participants*					0.906		
Orthodox Christian	46(48.9)	48(51.1)	94	1.1(0.6-2.1)	0.736		
Protestant Christian	132(48.5)	140(51.5)	272	1.1(0.6-2.0)	0.657		
Muslim	29(51.8)	27(48. 2)	56	1			

*Place of residence*					0.001**∗**		0.013**∗∗**
Urban	123(70.3)	52(29.7)	175	1		1	
Rural	84(34.0)	163(66.0)	247	4.5(3.0-6.9)	0.001	2.0(1.2 3.5)	0.013**∗∗**

*Ethnicity of the study participants*					0.364		
Gedeo	120(48.6)	127(51.4)	247	1.3(0.5-3.5)	0.569		
Oromo	34(41.5)	48(58.5)	82	1.7(0.6-4.9)	0.279		
Amhara	20(55.6)	16(44.4)	36	1.0(0.3-3.1)	1.000		
Sedama	23(59.0)	16(41.0)	39	0.8(0.3-2.7)	0.808		
Others	10(55.6)	8(44.4)	18	1			

*Occupation of the study participants*					0.915		
Farmer	92 (47.2)	103(52.8)	195	1.3(0.7-2.4)	0.462		
Employed	28(50.0 )	28(50.0 )	56	1.2(0.5-2.4)	0.754		
Daily laborer	21(46.7 )	24(53.3 )	45	1.3(0.6-2.9)	0.536		
Student	40(51.9)	37(48.1)	77	1.1(0.5-2.1)	0.903		
Merchant	26(53.1)	23(46.9)	49	1			

*Mean family size of the study participants*					0.099*∗*		
1-4 individuals	77(52.7)	69(47.3)	146	1		1	0.705
5-7 individuals	102(50.5)	100(49.5)	202	1.1(0.7-1.7)	0.679	1.2(0.7-2.2)	0.482
>7 individuals	28(37.8)	46 (62.2 )	74	1.8(1.0-3.2)	0.038	1.3(0.6-2.9)	0.481

*Estimated mean monthly family income/birr*					0.016*∗*		0.929
≤500	70(40.7)	102(59.3)	172	1.8(0.9-3.3)	0.074	1.1(0.4 2.9)	0.783
501-1000	72(59 )	50( 41)	122	0.8(0.4-1.6)	0.618	0.9(0.4-2.5)	0.926
>1000	28(54.9)	23(45.1)	51	1		1	
Unknown/ No- regular income	37( 48.1)	40(51.9)	77	1.3(0.6-2.7)	0.448	0.9(0.3-2.7)	0.853

*Presence of previous history of TB*					0.247*∗*		
Yes	21(58.3)	15(41.7)	36	1			
No	186(48.2)	200(51.8)	386	1.5(0.7-3.0)	0.247		

*Level of severity before first visit to health facility*					0.001**∗**		0.379
Still do a full day work	72(59.5)	49(40.5)	121	1		1	
Some activity outside the house	103(54.5)	86(45.5)	189	1.2(0.8-1.9)	0.386	1.4(0.7-2.6)	0.311
House/bed bound	32(28.6)	80(71.4)	112	3.7(2.1-6.3)	0.001	1.7(0.8 3.7)	0.174

*Smoking status/behaviors of the participants*					0.561		
Never smoke	146(50.3)	144(49.7)	290	1			
Former smoker	34(49.3)	35(50.7)	69	1.3(0.8 2.3)	0.282		
Current smoker	27(42.9)	36(57.1)	63	1.1(0.6-1.7)	0.873		

*Alcoholic intake status/behaviors of the study participants*					0.711		
Never drink	102(48.8)	107(51.2)	209	1			
Former drinker	67(51.5)	63(48.5)	130	1.1(0.7-1.8)	0.641		
Current drinking	38(45.8)	45(54.2)	83	0.9(0.6-1.4)	0.624		

*Knowledge score to TB*					0.001**∗**		0.038**∗∗**
Good	140(59.8)	94(40.2)	234	1		1	
Poor	67(35.6)	121(64.4)	188	2.7(1.8-3.9)	0.001	1.8(1.0-3.2)	0.038**∗∗**

*Stigma score to TB*					0.123**∗**		0.931
No/mild	132(53.0)	117(47.0)	249	1		1	
Moderate	47(41.6)	66(58.4)	113	1.6(1.0-2.5)	0.045	0.9(0.5 1.8)	0.862
Sever	28(46.7)	32(53.3)	60	1.3(0.7-2.3)	0.378	0.8(0.4 1.9)	0.714

*Patient satisfaction score to healthcare system*					0.757		
Good	87(49.7)	88(50.3)	175	1			
Medium	110(49.3)	113(50.7)	223	1.0(0.7-1.5)	0.939		
Sever	10(41.7)	14(58.3)	24	1.4(0.6-3.3)	0.461		

*Home distance from the nearest health facility*					0.001**∗**		0.005**∗∗**
≤10km	149(64.2)	83(35.8)	232	1		1	
>10km	58(30.5)	132(69.5)	190	4.1(2.7-6.1)	0.001	2.2(1.3-3.7)	0.005**∗∗**

*Presence of other HF close to home*					0.001**∗**		0.123
Yes	113(64.6)	62(35.4)	175	1			
No	94(38.1)	153(61.9)	247	2.9(1.9-4.4)	0.001	1.5(0.9-2.7)	0.123

*First health care consultation action because of the illness*					0.001**∗**		0.002**∗∗**
Gov.t health facility	107(67.3)	52(32.7)	159	1		1	
Formal private health provider	80(52.6)	72(47.4)	152	1.8(1.2- 2.9)	0.009	1.6(0.9-2.9)	0.082
Nonformal health provider	20(18.0)	91(82.0)	111	9.4(5.2-16.8)	0.001	3.8(1.8-7.9)	0.001**∗∗**

*Number healthcare providers visited*					0.001**∗**		0.184
≤2 providers	169(59.9)	113(40.1)	282	1		1	
≥3 providers	38(27.1)	102(72.9)	140	4.0(2.6-6.2)	0.001	1.5(0.8-2.9)	0.184

*Antibiotic Rx because of current illness*					0.001**∗**		0.001**∗∗**
Yes	78(35.6)	141(64.4)	219	3.1(2.1- 4.7)	0.001	6.5(3.6-11.7)	0.001**∗∗**
No	129(63.5)	74(36.5)	203	1		1	

*Keys*: *∗* P value ≤ 0.25 in bivariate analysis, *∗∗* P value ≤ 0.05 in multivariate analysis and its significantly independent associated factors.

## Data Availability

The “data collection Questionnaire” data used to support the findings of this study are included within the supplementary information file.

## Supplementary Materials

Supplementary Materials Questionnaire to assessment of total delay in treatment of TB and associated factors among new pulmonary TB patients in selected health facilities of Gedeo Zone, Southern Ethiopia, 2017/1.
