# Supplementary material for: Total Delay in Treatment of Tuberculosis and Associated Factors among New Pulmonary TB Patients in Selected Health Facilities of Gedeo Zone, Southern Ethiopia, 2017/18
Source: Interdiscip Perspect Infect Dis. 2019 Jun 2;2019:2154240. doi: 10.1155/2019/2154240 (PMC6582841; doi:10.1155/2019/2154240)
Supplement: Supplementary Materials — Questionnaire to assessment of total delay in treatment of TB and associated factors among new pulmonary TB patients in selected health facilities of Gedeo Zone, Southern Ethiopia, 2017/1. [file 2154240.f1.zip › 2154240/Data collection tool_IPID_2761333.docx]

**[Data collection tool](file:///D:\\1.%20Dilla%20university%20%20file\\staff%20research%20file\\Delay%20in%20initiating%20tuberculosis%20treatment\\final\\29)**

Questionnaire to assessment of total delay in treatment of TB and associated factors among new pulmonary TB patients in selected health facilities of Gedeo Zone, Southern Ethiopia, 2017/18

Patient ID. No_: ____________

**Instruction**: Please encircle the answer of the study participates in the question item.

| S. No. | **Questions** | **Question Items/category** | **Code** |
| --- | --- | --- | --- |
|  | **Part-I: socio-demographic information** | | |
| 101 | Age (year) | _________ |  |
| 102 | Gender | 1. Male 2. Female |  |
| 103 | Marital status | 1. Married 2. Single  3. Other (Specify)_______ |  |
| 104 | Educational status | 1. Not attended formal education  2. Primary school 3. Secondary school  4. Higher school |  |
| 105 | Religion | 1. Orthodox 2. Protestant  3. Muslim 4. Others (specify)____ |  |
| 106 | Place of residence | 1. Urban 2. Rural  3. Others (specify)_____ |  |
| 107 | Ethnicity | 1. Gedeo 2. Oromo 3. Amhara  4. Sedama 6. Other (specify)___ |  |
| 108 | Occupation | 1. Farmer 2. Employed  3. Daily Laborer 4. Student  5. Merchant 6. Other (Specify)____ |  |
| 109 | Family monthly income (ETB) | ________ |  |
| 110 | Mean family size in household | ________ |  |
| **Part - II: Co-morbidity & behavioral related factors** | | | |
| 201 | Presence of Previous history of TB | 1. Yes 2. No |  |
| 202  204 | Severity of the current disease upon first presentation to health facility | 1. Still do a full day’s work  2. Some activities outside the house  3. House or bed-bound  4. Others (specify)_____ |  |
| 205 | Smoker | 1. Never smoke 2. Current smoke  3. Former smoker |  |
| 206 | Alcohol intake | 1. Never drink 2. Current drinking  3. Former drinker |  |
| **Part-III: Health care accessibility& Health seeking behavior** | | | |
| 301 | How far is your home from this health facility? | _________ km |  |
| 302 | Is there other health facility closer to your home? | 1. Yes 2. No |  |
| 303 | Do you have visited other health facility prior to this health facilities contact? | 1. Yes 2. No |  |
| 304 | What type of health facility do you consult first because current illness? | 1. Government health care providers 2.Formal Private health care providers  3. Non- Formal Private health care providers |  |
| 305 | Number of health care provider visited until the current diagnosis made? | ____________ |  |
| 305 | Did you seek any treatment (antibiotics) from somewhere else because of the first symptom before this diagnosis? | 1. Yes 2. No |  |
| **Part -IV: Estimation of delays** | | | |
| 401 | How long did you sick starting from onset of TB symptoms until first sought treatment in this health facilities? | _____ days *(Total Treatment delay)* |  |
| **Part-V: Knowledge related factors** | | | |
| 501 | Do you know what TB is? | 1. Yes 2. No |  |
| 502 | Do you think that TB a serious disease? | 1. Yes 2. No |  |
| 503 | What in your own opinion causes TB? | 1. Infection 2. Punishment  3. Unavoidable 4. Don't know  5. Others (specify)_______ |  |
| 504 | What are the symptoms of someone infected with TB? | 1. Cough for more than 2 weeks  2. Sputum with blood 3. Fever  4. Weight loss 5. Don't know |  |
| 505 | Do you believe that TB is caused by mycobacterium TB? | 1. Yes 2. No |  |
| 506 | Do you think tuberculosis contagious? | 1. Yes 2. No |  |
| 507 | Do you believe that TB transmitted by air? | 1. Yes 2. No |  |
| 508 | Do you believed that TB is transmitted by hereditary? | 1. Yes 2. No |  |
| 509 | Do you believe that keeping away from the infected individual can prevent TB transmission? | 1. Yes 2. No |  |
| 510 | Do you know how TB is diagnosed? | 1. Yes 2. No |  |
| 511 | If Q No. 610 Yes, how it diagnosis? | 1. Sputum examination 2. X-ray 3. Other ___ |  |
| 512 | Do you know that TB is curable? | 1. Yes 2. No |  |
| 513 | Do you know what some cases of TB will require a longer treatment to be cured? | 1. Yes 2. No |  |
| 514 | Do you know that TB treatment is free? | 1. Yes 2. No |  |
|  | *Knowledge scores* | 1. Good 2. Poor |  |
| **Part-VI: Stigma related factors (**Please circle a score of 1= strongly disagree, 2= disagree, 3= agree, 4= strongly agree) to each questions. | | | |
| 601 | Do you think TB patients should be helped with free medicine & transportation to access to the health facility? |  |  |
| 602 | Do you believe that people with TB disclose their illness to other people? | 1 2 3 4 5 |  |
| 603 | Do you feel that TB can affect your relation with others such as families or friends? | 1 2 3 4 5 |  |
| 604 | Do you feel alone after discovering that you have TB within the community? | 11 2 3 4 5 |  |
| 605 | Do you feel ashamed of your TB disease? | 1 2 3 4 5 |  |
| 706 | Have you been afraid you may lose your job if it is known you have TB? | 1 2 3 4 5 |  |
| 607 | Do you think female are more discriminated against TB than male patients? | 1 2 3 4 5 |  |
| 608 | Do you think that TB will affect the ability to become pregnant/ have healthy children in female? | 1 2 3 4 5 |  |
| 609 | Do you need permission from your family or relatives to access health services? | 1 2 3 4 5 |  |
| 610 | Is there less chance of marriage due to TB diagnosis? | 1 2 3 4 5 |  |
| 611 | *Stigma scores* | 1. No stigma/mild 2. Moderate  3. Sever stigma |  |
| **Part-VII: Patient Satisfaction/feedback** on health care providers & health care system on previous service **(**Please circle a score of 1= very low, 2= low, 3= medium, 4= good, 5= very good) to each questions | | | |
| 701 | Are the health care providers supportive and respectful of people? | 1 2 3 4 5 |  |
| 702 | Before coming for diagnosis/ treatment, did you expect that the providers would be supportive and respectful to you? | 1 2 3 4 5 |  |
| 703 | Do most people in your community believe they will be treated supportively and respectfully by health care providers if they have TB? | 1 2 3 4 5 |  |
| 704 | Are you happy with the service you are getting from this facility? | 1 2 3 4 5 |  |
| 705 | What Is the level of service you are getting from this facility? | 1 2 3 4 5 |  |
| 706 | Have health care workers answered all of your questions concerning illness? | 1 2 3 4 5 |  |
| 707 | Are you satisfied with treatment received from other health workers? | 1 2 3 4 5 |  |
| 708 | Are you satisfied with clinic schedule? |  |  |
| 709 | Are you satisfied with waiting time in the clinic? | 1 2 3 4 5 |  |
| 710 | Are you satisfied with cost of consultation? | 1 2 3 4 5 |  |
| 711 | Are you satisfied with cost of treatment? | 1 2 3 4 5 |  |
| 8712 | Are you satisfied with availability of drugs at the health center? | 1 2 3 4 5 |  |
| 713 | Are you satisfied with you treatment partner/ DOTS supporter | 1 2 3 4 5 |  |
|  | ***Patient Satisfaction score*** | 1. Good, 2. Medium 3. Low |  |
