# Supplementary material for: Total Delay in Treatment of Tuberculosis and Associated Factors among New Pulmonary TB Patients in Selected Health Facilities of Gedeo Zone, Southern Ethiopia, 2017/18
Source: Interdiscip Perspect Infect Dis. 2019 Jun 2;2019:2154240. doi: 10.1155/2019/2154240 (PMC6582841; doi:10.1155/2019/2154240)
Supplement: Supplementary Materials — Questionnaire to assessment of total delay in treatment of TB and associated factors among new pulmonary TB patients in selected health facilities of Gedeo Zone, Southern Ethiopia, 2017/1. [file 2154240.f1.zip › 2154240/Data collection tool_IPID_2761334.pdf]

Data collection tool

Questionnaire to assessment of total delay in treatment of TB and associated factors among new pulmonary TB patients in selected health facilities of Gedeo Zone, Southern Ethiopia, 2017/18

Patient ID. No_: ____________

Instruction: Please encircle the answer of the study participates in the question item.

S. No.	Questions	Question Items/category	Code
	**Part-I: socio-demographic information**		
101	Age (year)	__________	
102	Gender	1. Male 2. Female	
103	Marital status	1. Married 2. Single<br>3. Other (Specify)_______	
104	Educational status	1. Not attended formal education<br>2. Primary school 3. Secondary school<br>4. Higher school	
105	Religion	1. Orthodox 2. Protestant<br>3. Muslim 4. Others (specify)____	
106	Place of residence	1. Urban 2. Rural<br>3. Others (specify)_____	
107	Ethnicity	1. Gedeo 2. Oromo 3. Amhara<br>4. Sedama 6. Other (specify)___	
108	Occupation	1. Farmer 2. Employed<br>3. Daily Laborer 4. Student<br>5. Merchant 6. Other (Specify)____	
109	Family monthly income (ETB)	________	
110	Mean family size in household	________	
**Part - II: Co-morbidity & behavioral related factors**			
201	Presence of Previous history of TB	1. Yes 2. No	
202<br>204	Severity of the current disease upon first presentation to health facility	1. Still do a full day's work<br>2. Some activities outside the house<br>3. House or bed-bound<br>4. Others (specify)_____	
205	Smoker	1. Never smoke 2. Current smoke<br>3. Former smoker	
206	Alcohol intake	1. Never drink 2. Current drinking<br>3. Former drinker	
**Part-III: Health care accessibility& Health seeking behavior**			
301	How far is your home from this health facility?	_________ km	
302	Is there other health facility closer to your home?	1. Yes 2. No	

303	Do you have visited other health facility prior to this health facilities contact?	1. Yes 2. No	
304	What type of health facility do you consult first because current illness?	1. Government health care providers<br>2.Formal Private health care providers<br>3. Non- Formal Private health care providers	
305	Number of health care provider visited until the current diagnosis made?	____________	
305	Did you seek any treatment (antibiotics) from somewhere else because of the first symptom before this diagnosis?	1. Yes 2. No	
**Part -IV: Estimation of delays**			
401	How long did you sick starting from onset of TB symptoms until first sought treatment in this health facilities?	_____ days *(Total Treatment delay)*	
**Part-V: Knowledge related factors**			
501	Do you know what TB is?	1. Yes 2. No	
502	Do you think that TB a serious disease?	1. Yes 2. No	
503	What in your own opinion causes TB?	1. Infection 2. Punishment<br>3. Unavoidable 4. Don't know<br>5. Others (specify)_______	
504	What are the symptoms of someone infected with TB?	1. Cough for more than 2 weeks<br>2. Sputum with blood 3. Fever<br>4. Weight loss 5. Don't know	
505	Do you believe that TB is caused by mycobacterium TB?	1. Yes 2. No	
506	Do you think tuberculosis contagious?	1. Yes 2. No	
507	Do you believe that TB transmitted by air?	1. Yes 2. No	
508	Do you believed that TB is transmitted by hereditary?	1. Yes 2. No	
509	Do you believe that keeping away from the infected individual can prevent TB transmission?	1. Yes 2. No	
510	Do you know how TB is diagnosed?	1. Yes 2. No	
511	If Q No. 610 Yes, how it diagnosis?	1. Sputum examination 2. X-ray 3. Other ___	
512	Do you know that TB is curable?	1. Yes 2. No	
513	Do you know what some cases of TB will require a longer treatment to be cured?	1. Yes 2. No	
514	Do you know that TB treatment is free?	1. Yes 2. No	
	Knowledge scores	1. Good 2. Poor	

**Part-VI: Stigma related factors (**Please circle a score of 1= strongly disagree, 2= disagree, 3= agree, 4= strongly agree) to each questions.			
601	Do you think TB patients should be helped with free medicine & transportation to access to the health facility?		
602	Do you believe that people with TB disclose their illness to other people?	1 2 3 4 5	
603	Do you feel that TB can affect your relation with others such as families or friends?	1 2 3 4 5	
604	Do you feel alone after discovering that you have TB within the community?	11 2 3 4 5	
605	Do you feel ashamed of your TB disease?	1 2 3 4 5	
706	Have you been afraid you may lose your job if it is known you have TB?	1 2 3 4 5	
607	Do you think female are more discriminated against TB than male patients?	1 2 3 4 5	
608	Do you think that TB will affect the ability to become pregnant/ have healthy children in female?	1 2 3 4 5	
609	Do you need permission from your family or relatives to access health services?	1 2 3 4 5	
610	Is there less chance of marriage due to TB diagnosis?	1 2 3 4 5	
611	*Stigma scores*	1. No stigma/mild 2. Moderate<br>3. Sever stigma	
**Part-VII: Patient Satisfaction/feedback** on health care providers & health care system on previous service **(**Please circle a score of 1= very low, 2= low, 3= medium, 4= good, 5= very good) to each questions			
701	Are the health care providers supportive and respectful of people?	1 2 3 4 5	
702	Before coming for diagnosis/ treatment, did you expect that the providers would be supportive and respectful to you?	1 2 3 4 5	
703	Do most people in your community believe they will be treated supportively and respectfully by health care providers if they have TB?	1 2 3 4 5	
704	Are you happy with the service you are getting from this facility?	1 2 3 4 5	

705	What Is the level of service you are getting from this facility?	1	2	3	4	5	
706	Have health care workers answered all of your questions concerning illness?	1	2	3	4	5	
707	Are you satisfied with treatment received from other health workers?	1	2	3	4	5	
708	Are you satisfied with clinic schedule?						
709	Are you satisfied with waiting time in the clinic?	1	2	3	4	5	
710	Are you satisfied with cost of consultation?	1	2	3	4	5	
711	Are you satisfied with cost of treatment?	1	2	3	4	5	
8712	Are you satisfied with availability of drugs at the health center?	1	2	3	4	5	
713	Are you satisfied with you treatment partner/ DOTS supporter	1	2	3	4	5	
	Patient Satisfaction score	1. Good,	2. Medium	3. Low			
